# Development and Validation of the Children’s Emotions Database (CED): Preschoolers’ Basic and Complex Facial Expressions

**DOI:** 10.3390/children12070816

**Published:** 2025-06-21

**Authors:** Nadia Koltcheva, Ivo D. Popivanov

**Affiliations:** 1 Department of Cognitive Science and Psychology, New Bulgarian University, 1618 Sofia, Bulgaria; ipopivanov@nbu.bg; 2 Clinic of Neurology, University Hospital “Alexandrovska”, 1431 Sofia, Bulgaria

**Keywords:** emotions, emotion recognition, Children’s Emotions Database (CED), preschoolers, facial expressions, complex emotions

## Abstract

**Background**. Emotions are a crucial part of our human nature. The recognition of emotions is an essential component of our social and emotional skills. Facial expressions serve as a key element in discerning others’ emotions. Different databases of images of facial emotion expressions exist worldwide; however, most of them are limited to only adult faces and include only the six basic emotions, as well as neutral faces, ignoring more complex emotional expressions. Here, we present the Children’s Emotions Database (CED), a novel repository featuring both basic and complex facial expressions captured from preschool-aged children. The CED is one of the first databases to include complex emotional expressions in preschoolers. Our aim was to develop such a database that can be used further for research and applied purposes. **Methods.** Three 6-year-old children (one female) were photographed while showing different facial emotional expressions. The photos were taken under standardized conditions. The children were instructed to express each of the following basic emotions: happiness, pleasant surprise, sadness, fear, anger, disgust; a neutral face; and four complex emotions: pride, guilt, compassion, and shame; this resulted in a total of eleven expressions for each child. Two photos per child were reviewed and selected for validation. The photo validation was performed with a sample of 104 adult raters (94 females; aged 19–70 years; M = 29.9; SD = 11.40) and a limited sample of 32 children at preschool age (17 girls; aged 4–7 years; M = 6.5; SD = 0.81). The validation consisted of two tasks—free emotion labeling and emotion recognition (with predefined labels). Recognition accuracy for each expression was calculated. **Results and Conclusions.** While basic emotions and neutral expressions were recognized with high accuracy, complex emotions were less accurately identified, consistent with the existing literature on the developmental challenges in recognizing such emotions. The current work is a promising new database of preschoolers’ facial expressions consisting of both basic and complex emotions. This database offers a valuable resource for advancing research in emotional development, educational interventions, and clinical applications tailored to early childhood.

## 1. Introduction

Emotions are a crucial aspect of human nature, serving as a cornerstone of social interaction, communication, and personal development. The ability to recognize and interpret emotions accurately is an essential component of social and emotional competence. Among the various modalities through which emotions are expressed, facial expressions are especially significant for discerning others’ emotional states. From infancy, humans exhibit sensitivity to facial emotional cues, highlighting the evolutionary and developmental importance of emotion recognition [1].

- **Theoretical Framework**

The importance of both basic and complex emotional expressions is supported by theoretical models such as Ekman’s Neurocultural Theory of Emotion [2] and the developmental model of emotion recognition [3]. According to these frameworks, emotional expression is influenced by both biologically innate mechanisms and social learning processes. A database focused specifically on preschool-aged children’s facial expressions, including complex emotions, would provide critical empirical data for testing and refining these theories. It would also shed light on the developmental trajectories of emotional competence and the interplay between innate and culturally mediated emotional processes in early childhood.

The conceptual foundation for this study rests on Ekman’s Neurocultural Theory of Emotion (1972) [2], which proposes that certain basic emotions are universally expressed and recognized due to biological underpinnings, while more complex emotions require social learning. This aligns with developmental models such as Camras and Allison’s (1985) [3], which suggest that while infants and toddlers can recognize and produce basic emotions, complex emotions like guilt or pride emerge only as children gain cognitive maturity and social awareness.

These complex emotions are often shaped by internalized moral codes, self-consciousness, and the understanding of social rules—processes that develop during the preschool years. Izard’s Differential Emotions Theory [4] also supports this trajectory, emphasizing that emotional development is intertwined with growing cognitive and social capacities. A database reflecting these developmental shifts is thus essential for research and intervention efforts targeting early emotional competence.

- **Development of Basic Emotion Recognition in Children and Adults**

The ability to recognize facial expressions of emotion begins early in life and continues to develop throughout childhood and adolescence. Infants as young as a few months old can discriminate between happy and sad faces, and by the end of the first year, they begin to respond differentially to a wider range of emotional expressions [5,6]. During the preschool years, children’s recognition accuracy for basic emotions—such as happiness, sadness, anger, and fear—improves significantly as their social experiences and language skills expand [7,8]. The recognition of emotions like disgust and surprise typically emerges later due to their more nuanced visual cues. In contrast, adults generally show high accuracy in recognizing all basic emotions, which is attributed to cumulative social learning and cognitive maturity [9,10]. However, this developmental progression is influenced by various factors, including culture, context, familiarity with the expresser, and task format [11]. Understanding these developmental trajectories is essential for creating and validating age-appropriate emotional expression databases.

Building on Darwin’s foundational work on emotional expression (Darwin, 1872; 2015, as cited in [1,2]) and the identification of six universally recognized basic emotions (happiness, sadness, fear, disgust, surprise, and anger), subsequent research has consistently emphasized the centrality of facial expressions in emotional cognition and social adaptation.

- **Existing Databases**

Over the past decades, numerous facial expression databases have been developed, primarily featuring adult models and basic emotions (e.g., Ekman & Friesen, 1971, 1975 [12,13]; Tottenham et al., 2009 [14]). The availability of standardized facial expression databases has significantly advanced research on emotion recognition. These databases typically contain static images or video sequences of individuals displaying various emotions under controlled conditions. They have enabled investigations across multiple domains, ranging from basic psychological research to applications in affective computing and clinical practice [1]. However, the majority of existing databases feature adult actors and are primarily based on posed, rather than naturally elicited, expressions, thus limiting their ecological validity and relevance for developmental research.

A systematic review by Fabricio and colleagues (2022) [1] identified 36 such databases, highlighting significant heterogeneity in the methodologies used to elicit and validate emotional expressions. Most utilize adult participants whose emotional expressions are induced in semi-controlled settings to enhance spontaneity. Stimuli are predominantly static, colorful images captured against standardized backgrounds, with standardized clothing to minimize extraneous variables. Despite these efforts, substantial variation remains in the methods used to induce, capture, and validate emotional expressions, potentially affecting the comparability and generalizability of findings across studies and populations.

While facial emotion recognition has been widely studied in adults using databases like Ekman’s Pictures of Facial Affect or the NimStim set [13], emotion expression databases for children—especially preschoolers—remain scarce and limited. Relying on Fabricio and colleagues’ (2022) [1] systematic review, we were able to identify only five databases, some of which hardly match our target age (Table 1). The scope of all of them is restricted to basic emotions and does not include complex emotions such as pride, guilt, or compassion.

- **Need for Preschooler Databases**

A critical gap exists in the representation of children’s facial expressions, particularly those of preschool-aged children. While a few databases, such as the Dartmouth Database of Children’s Faces and the Picture-Set of Young Children’s Affective Facial Expressions (PSYCAFE) [15,16], include young participants, they remain limited in scope, emotional range, and cultural diversity. Most notably, existing child-focused databases predominantly depict basic emotions and often overlook complex emotional states such as pride, guilt, compassion, and shame.

Understanding complex emotions is vital because they play a significant role in socialization, moral development, and interpersonal relationships in early childhood. Unlike basic emotions, complex emotions involve self-reflection, social context, and internalized standards, making them critical markers of socio-emotional and cognitive maturity. Their accurate recognition and expression are associated with school readiness, peer acceptance, and long-term mental health outcomes. The lack of available stimuli depicting complex emotions among preschool-aged children thus impedes a comprehensive understanding of early emotional development and limits the effectiveness of diagnostic and educational interventions.

The development of the Children’s Emotions Database (CED) was motivated by the limitations of existing emotional expression sets. While widely used databases such as the Pictures of Facial Affect (POFA; [13]) and NimStim [14] offer well-controlled images of adult models displaying basic emotions, they are not suitable for studies involving children—either as expressers or as target perceivers. Even among child-focused databases, including the Dartmouth Database [15], the Child Affective Facial Expression set (CAFE; [17]), and Child Emotions Picture Set [19], consist of expressions of basic emotions such as happiness, sadness, anger, fear, and surprise, and predominantly include school-aged children or adolescents.

This presents a significant developmental gap, as few databases focus specifically on preschool-aged children, despite this being a key period for the acquisition of emotional understanding, vocabulary, and regulation strategies. Children between the ages of 3 and 7 experience rapid growth in their socio-emotional competence, yet their emotional expressions—especially more complex, socially constructed emotions—are underrepresented in available resources. Most existing databases lack depictions of nuanced emotional states such as guilt, pride, or compassion, which are essential for studying the emergence of moral reasoning, empathy, and interpersonal behavior in early childhood.

The CED was specifically designed to address these limitations by including a diverse sample of preschoolers and capturing both basic and complex emotional expressions. By introducing a validated set of facial expressions across a broader emotional range, the CED provides a novel tool for researchers, educators, and clinicians to investigate emotion recognition, regulation, and expression in early childhood with greater sensitivity and developmental relevance.

- **Aim of the Present Work**

In response to these gaps, the current project aimed to develop and validate the Children’s Emotions Database (CED), a novel repository of facial expressions featuring both basic and complex emotions in preschool-aged children. By addressing key limitations of existing databases, the CED offers a valuable resource for researchers, educators, and clinicians. It holds the potential to enhance the ecological validity of studies in emotional development, support the creation of developmentally appropriate assessment tools, and inform interventions aimed at fostering children’s social and emotional skills.

## 2. Materials and Methods

### 2.1. Participants in the Database

Three 6–7-year-old children (M = 6.86; SD = 0.29) of Bulgarian origin (two boys and one girl) took part in the photoshoot. All children were members of theater school for at least one year. They participated voluntarily and their parents signed an informed consent form. The study was approved by the Ethical Committee of the Department of Cognitive Science and Psychology, NBU (No. 647/12 September 2023).

### 2.2. Stimuli Development Procedure

**Figure 1:**
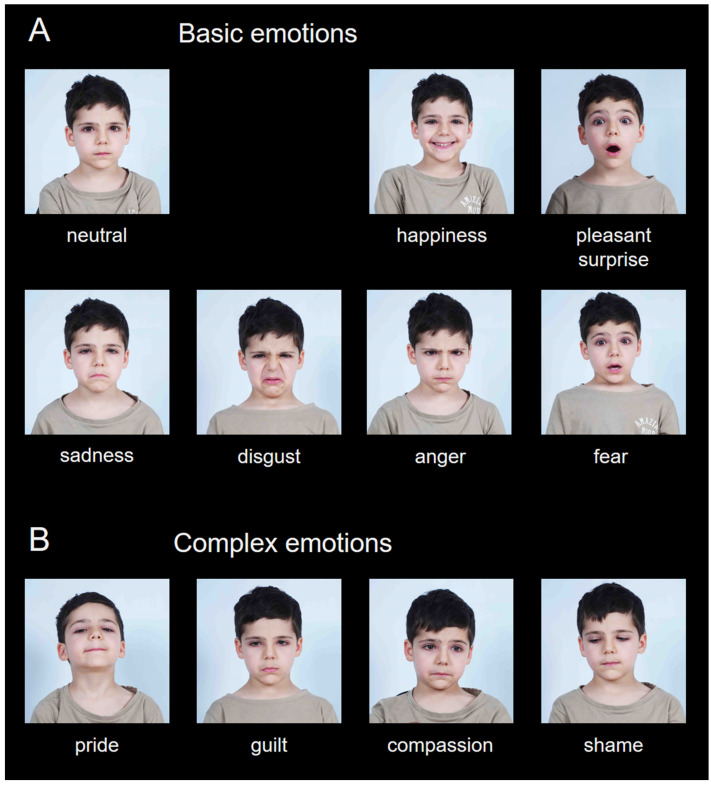
Examples of the facial emotional expressions of one of the children in CED. (**A**) Six basic facial emotional expressions together with a neutral facial expression. (**B**) Four complex facial emotional expressions.

Frontal color portrait photos of the children were taken with a Sony ZV-1 20MPx digital camera under standardized conditions (see Figure 1). The camera was positioned approximately 1.5 m away from the actor. Two 1000 W UNOMAT LX901GZ video-lamps were placed at each side of the actors, ensuring uniform light distribution on the face. All children were photographed against the same white vinyl background, placed 50 cm behind the actor.

After a brief general instruction session, the children were asked to express each of the following 11 emotional states with their faces: joy, surprise (pleasant), sadness, fear, anger, disgust, a neutral face (basic emotions, Figure 1A), and pride, guilt, compassion, and shame (complex emotions, Figure 1B). When necessary, short situational stories were used to elicit more authentic expressions, e.g., *Imagine you have seen your best friend suffer because his pet is sick* (compassion) or *Imagine you have received a fantastic present for your birthday* (pleasant surprise).

Several photos were taken for each emotional state for each child. Then only a few photos (1–3) per child per emotion were selected by two experts resulting in total of 60 photos (6 stimuli of happiness, 5 of pleasant surprise, 6 of pride, 5 of neutral expression, 6 of compassion, 6 of anger, 6 of fear, 6 of sadness, 5 of disgust, 6 of shame, and 3 of guilt). After the photos were taken, they were cropped and saved in JPEG format with a size of 1050 × 1050 px, a resolution of 96 dpi, and a bit depth of 24. As such, the images were ready for online validation procedure.

### 2.3. Validation of the Children’s Emotions Database with Adult Subjects

#### 2.3.1. Participants for Validation

In total, 104 Bulgarian volunteers (94 women), aged between 19 and 70 years (M = 29.9 SD = 11.40), took part in the online validation. Some of them were students at NBU, who participated in exchange for a course credit, and others were volunteers recruited through social networks.

#### 2.3.2. Validation Procedure

The validation procedure took place online using the Google Forms platform. The participants had to perform two separate tasks with all the photos. In both tasks, the photos were shown one at a time in a randomized order.

The first task was the free naming of the emotion depicted in the picture. The participants were allowed to give a short answer (a word or a phrase) in a free written form. The second task was an emotion recognition task with 11 predefined labels—the same as the ones shown in Figure 1.

- **Task 1: Free Emotion Naming**
  Participants were instructed to provide a free-text label (a word or short phrase) that best described the emotion depicted in each photograph.
- **Task 2: Emotion Recognition with Predefined Labels**
  Participants were asked to identify the emotion displayed in each photograph by selecting from 11 predefined emotion labels corresponding to the intended expressions (i.e., the 7 basic and 4 complex emotions).

Each task was completed independently to avoid bias. No feedback was provided between tasks.

### 2.4. Validation of the Children’s Emotions Database with Preschool Children Subjects

#### 2.4.1. Participants for Validation

We were able to collect responses for the free naming and emotion recognition task from a limited sample of preschool children of Bulgarian origin. In total, 32 of them (17 girls), aged between 4 years and 9 months and 7 years and 11 months (M = 6 years and 6 months; SD = 9.7 months), took part in the online free naming task. In total, 28 of them (15 girls), aged between 4 years and 11 months and 7 years and 11 months (M = 6 years and 10 months SD = 8.6 months), also took part in the online recognition task. The children’s parents filled in an informed consent form for their participation in the study.

#### 2.4.2. Validation Procedure

The validation procedure took place online using the Google Forms platform. The participants had to perform the same two tasks—free emotion naming and emotion recognition with predefined labels—as described in Section 2.3.2. Since most of the participants were not able to read, their parents read the instructions and the possible choices (in the recognition task) and filled in their answers.

Each task was completed independently, with a short break between them. No feedback was provided between tasks.

## 3. Results

To evaluate the accuracy of emotion naming and recognition across age groups, we used descriptive statistics (mean percent correct responses) and inferential analyses. For comparisons between adult and child participants, we performed a series of 2 × 2 Chi-square (χ^2^) tests on each emotional expression (group: adults vs. children × response: correct vs. incorrect). In addition, we compared confusion patterns using confusion matrices.

### 3.1. Accuracy of Emotion Naming and Recognition—Adults

Recognition accuracy for each emotional expression was assessed separately for the free naming and predefined-label tasks.

**Figure 2:**
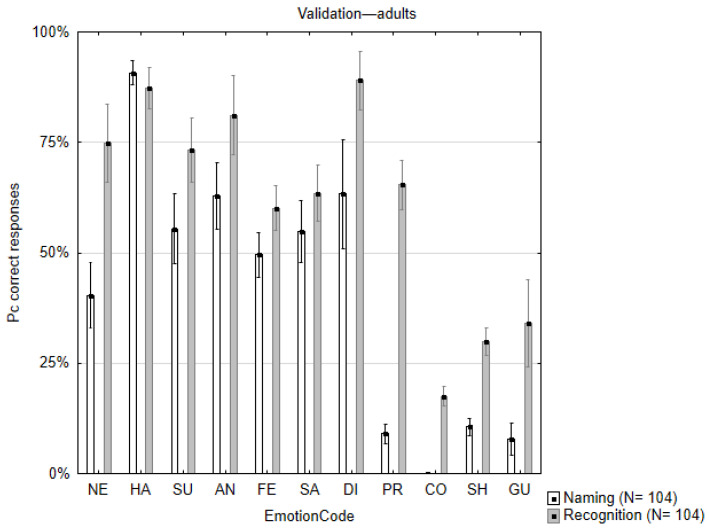
Averaged percent correct responses for each of the eleven facial emotional expressions in the free naming (white bars) and the recognition (gray bars) validation tasks with adult participants. Note the relatively lower correct responses for the complex emotions. Vertical bars denote SEM. Emotion codes: HA—Happiness; SU—Surprise; PR—Pride; NE—Neutral; CO—Compassion; AN—Anger; FE—Fear; SA—Sadness; DI—Disgust; SH—Shame; GU—Guilt.

For the free naming task, two experts (the co-authors) reviewed all the answers. The answers were then classified as *correct* (correctly named emotion or its synonym) or *incorrect* (incorrectly named emotion). The percent correct responses for each stimulus were counted and then averaged across the 11 emotional expressions (see column “Naming” in Table A1 and white bars in Figure 2).

For the emotion recognition task, first the correct answers were counted for each stimulus. Next, the percent correct responses were averaged across the 11 emotional expressions. The results are summarized in Table A1 (column “Recognition”) and visualized in Figure 2 (gray bars).

All basic emotional expressions (happiness, surprise, sadness, fear, anger, and disgust) were recognized and named correctly by more than 50% of the participants. The only exception was the naming of the neutral expression, which was a bit lower than 50% correct. As expected, the percent correct responses were relatively higher in the recognition task for all emotional expressions (except for happiness). This was especially evident for the complex emotional expressions (pride, guilt, compassion, and shame) which had very low percent correct responses in the naming task.

### 3.2. Accuracy of Emotion Naming and Recognition—Children

**Figure 3:**
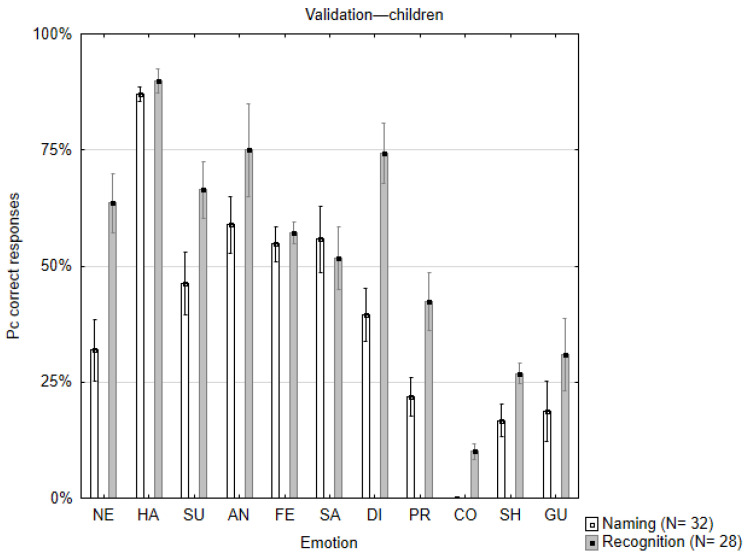
Averaged percent correct responses for each of the eleven facial emotional expressions in the free naming (white bars) and the recognition (gray bars) validation tasks with child participants. Note the relatively lower correct responses for the complex emotions. Vertical bars denote SEM. Emotion codes: same as in Figure 2.

The percent correct responses were calculated following an identical procedure to the one described in Section 3.1. Figure 3 (and Table A2) presents the results of both tasks.

### 3.3. Comparison Between Naming and Recognition Performance of Adults and Children

Adults’ and children’s accuracy in naming and recognizing emotions was compared by means of multiple 2 × 2 (adults vs. children × correct vs. incorrect responses) Chi-square tests for each of the 11 facial emotional expressions. Bonferroni correction for multiple comparisons was applied to the significance level, so that alpha was set to 0.005 (0.05/11).

The analysis revealed no statistically significant differences between adults and children in either the naming or recognition tasks for any of the individual emotional expressions (all *p*-values > 0.02). These results suggest that preschool-aged children are able to name and recognize both basic and some complex emotional expressions with a similar level of accuracy as adults. However, it is important to note that the sample size for the children’s group was limited, which may affect the power of the statistical tests and should be considered when interpreting the results.

### 3.4. Confusion Patterns Among Emotions—Adults and Children

**Figure 4:**
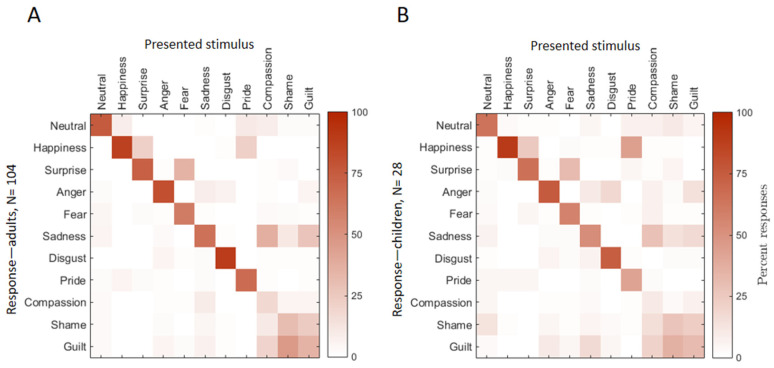
Confusion matrices based on the responses in the emotion recognition task of adult (**A**) and child participants (**B**). Each column represents 1 of the 11 presented emotional categories. Each row represents the responses out of the 11 possible labels. Note that complex emotions were confused much more than the basic ones.

Furthermore, we evaluated which of the emotional expressions were confused most often. To do so, we counted the percent correct responses for each expression as well as the percent responses to other expressions. Thus, we created confusion matrices with the presented expressions and responses given by the adult and child participants in the recognition task (Figure 4A,B and Table A3 and Table A4, respectively). These matrices present the proportion of responses for each presented emotion and reveal common patterns of confusion.

In Figure 4, it can be noted that in both tested samples, basic emotions and neutral expressions were recognized more reliably than the complex ones, as the latter ones were often confused with other expressions. For example, the expression of *compassion* was recognized more often as “sadness” and almost equally often as “guilt” in comparison to the correct response “compassion”. Similarly, expressions of *shame* and *guilt* were often confused between each other and also with the basic expression of “sadness”.

Additionally, our data replicates the well-known finding that the expression of *fear* can often be confused with “surprise”. Interestingly, the emotional expression of *surprise* was also recognized sometimes as “happiness”.

Finally, the confusion matrices of adults and children (Figure 4A,B, respectively) look remarkably similar, showing that children at preschool age are able to recognize basic and some complex emotions as well as adults. Thus, the CED seems to be a valid instrument for testing emotion recognition in preschool age.

Overall, basic emotions were recognized more reliably, showing higher percentages along the matrix diagonal (correct responses) compared to complex emotions. Complex emotions were frequently confused with both other complex emotions and some basic emotions.

## 4. Discussion and Conclusions

The present study introduces the Children’s Emotions Database (CED), a novel and valuable resource that addresses a significant gap in the field of developmental psychology. The CED captures both basic and complex emotional facial expressions in preschool-aged children, offering a more ecologically valid and developmentally appropriate tool than existing facial expression databases. By providing a validated set of emotional expressions from young children, the CED offers new opportunities for research, educational programming, and clinical assessments related to early childhood emotional development.

Our findings demonstrate that adults are more reliably able to recognize basic emotions, such as happiness, sadness, and anger, compared to complex emotions like pride, guilt, compassion, and shame. This pattern is consistent with established research in emotion recognition and emotional development, highlighting the developmental and cognitive challenges associated with understanding nuanced emotional states. The difficulty in recognizing complex emotions reflects the socially embedded nature of these feelings, emphasizing the need for further research to explore the developmental trajectory of emotion recognition in young children. Confusion matrices further indicated that participants tended to confuse complex emotions with each other or with related basic emotions such as sadness. These results underscore the inherent challenges associated with recognizing nuanced emotional states, especially when expressed by young children.

### 4.1. Theoretical Implications

These findings align with developmental theories suggesting that the ability to express and recognize basic emotions emerges early and is largely biologically driven [5], whereas the understanding of complex emotions develops later and is influenced more heavily by socialization and cultural learning [6]. The difficulty observed in recognizing complex emotions in preschool-aged children supports the notion that such emotions are still emerging during early childhood, both in their expression and their recognition by others.

Furthermore, the systematic confusion between certain emotions, such as fear and surprise, corroborates long-standing observations in emotion research, emphasizing that perceptual overlaps between certain expressions pose recognition challenges even for adults.

The relatively low recognition rates for complex emotions, such as compassion, likely reflect their higher cognitive demands. These emotions are socially constructed and involve internal states that may not manifest through prototypical facial expressions. This aligns with theories that suggest these emotions require mentalizing skills, such as perspective-taking.

Moreover, recognition accuracy was higher when participants selected from predefined categories compared to the free labeling task, suggesting that top–down processes (e.g., category priming) play a role in disambiguating children’s expressions. This has implications for designing emotion recognition tasks and underscores the importance of training both children and adults in recognizing complex emotional cues.

### 4.2. Practical Applications

The CED offers significant potential applications for research, education, and clinical practice. In research contexts, the database can facilitate more ecologically valid investigations into the development of emotion recognition abilities in both typical and atypical populations (e.g., children with autism spectrum disorder). In educational settings, the CED can inform the design of social–emotional learning (SEL) programs that aim to improve children’s emotional literacy by using age-relevant and realistic stimuli. Clinically, the database may aid in early identification of socio-emotional difficulties and inform intervention programs targeting emotional understanding and regulation.

### 4.3. Limitations

Several limitations of the present study warrant acknowledgment. The validation sample was predominantly female (approximately 91%), which may have influenced recognition patterns. Although emotion recognition is generally robust across genders, some research suggests subtle differences that future studies should examine more closely. The emotional expressions were elicited through instructed posing rather than spontaneous emotional reactions, which, although standardized, might not fully capture the dynamic and nuanced nature of natural emotional expression.

### 4.4. Future Directions

Future research should aim to expand the CED by testing emotion recognition among children, not only adults, which would provide important insights into developmental trajectories of emotional understanding. Investigating the impact of context (e.g., body language and situational background) alongside facial expressions would further enrich the ecological validity of emotion recognition research in young populations.

Future validation efforts should include child raters to assess developmental changes in peer emotion recognition. Peer-based evaluations are critical for educational and social intervention research and would improve the ecological validity of the database.

Additionally, cross-cultural validation is an essential next step. Collaborative studies across different cultural groups would allow the universality of both basic and complex emotion recognition to be tested. These expansions would strengthen the database’s utility across diverse populations and contexts.

### 4.5. Conclusions

The Children’s Emotions Database (CED) addresses a significant gap in the field of developmental psychology by providing a validated set of facial emotional expressions from preschool-aged children, including both basic and complex emotions. The CED represents an important advancement in the study of early emotional development. It provides a unique and promising tool for researchers, educators, and clinicians interested in understanding and supporting young children’s social and emotional growth. By offering a more comprehensive set of emotional expressions, the CED has the potential to inform the development of age-appropriate emotion recognition assessments, contribute to more effective social and emotional learning (SEL) programs, and aid in the early detection of developmental anomalies. As the database expands and future work addresses its limitations, it will continue to enrich our understanding of emotional development in early childhood, paving the way for advancements in emotion research and interventions.

## Figures and Tables

**Table 1:** Emotion expression databases with preschool children. (All the listed databases consist of six basic emotions (happiness, sadness, fear, disgust, anger, and surprise) and a neutral one.)

Database	Authors/Year	Age of the Models	Ethnicity	Emotions Included		Visual Characteristics/Stimuli
				Number	Six Basic and One Neutral	
The Dartmouth Database of Children’s Faces	Dalrymple et al. (2013) [15]	6–16 years	Caucasian	7	X	static, color
Picture-Set of Young Children’s Affective Facial Expressions (PSYCAFE)	Franz et al. (2021) [16]	4–6 years	NA	7	X	static, color
The Child Affective Facial Expression (CAFE)	LoBue and Thrasher (2015) [17]	2–8 years	African American, Asian, Caucasian/European American, Latino, South Asian	7	X	static, color
The Child Emotion Facial Expression Set	Negrão et al. (2021) [18]	4–6 years	Caucasian, African, Asian	7	X	static, color
Child Emotions Picture Set	Romani-Sponchiado et al. (2015) [19]	6–7, 8–9, and 10–11 years	Caucasian, Afro-American, indigenous	7	X	static, gray scale

## Data Availability

The data presented in this study are available on request from the corresponding author. The data are not publicly available due to the privacy.

## Abbreviations

The following abbreviations are used in this manuscript:

CED	Children’s Emotions Database
HA	Happiness
SU	Surprise
PR	Pride
NE	Neutral
CO	Compassion
AN	Anger
FE	Fear
SA	Sadness
DI	Disgust
SH	Shame
GU	Guilt
SEM	Standard Error of Mean

## Appendix A

The accuracy data from the two validation tasks with adult subjects are presented in Table A1.

**Table A1:** Proportion of correct responses in free naming task and emotion recognition task with predefined labels for each stimulus and averaged across emotion category. The data is based on the answers of N = 104 adult participants.

		Naming			Recognition		
Stimulus	Code	Pc Correct	M(Pc Correct)	SE(Pc Correct)	Pc Correct	M(Pc Correct)	SE(Pc Correct)
1_M1_HA_040	HA	0.90	0.91	0.03	0.74	0.87	0.05
1_M1_HA_056	HA	0.95			0.97		
1_M2_HA_282	HA	0.98			0.81		
1_M2_HA_281	HA	0.95			0.76		
1_F1_HA_161	HA	0.82			0.99		
1_F1_HA_162	HA	0.84			0.96		
1_MA_SU_094	SU	0.48	0.63	0.08	0.67	0.81	0.09
1_F1_SU_200	SU	0.65			0.91		
1_F1_SU_201	SU	0.41			0.64		
1_M2_SU_338	SU	0.40			0.89		
1_M2_SU_313	SU	0.82			0.54		
1_M1_PR_128	PR	0.04	0.09	0.02	0.45	0.65	0.06
1_M1_PR_130	PR	0.17			0.84		
1_F1_PR_208	PR	0.07			0.56		
1_F1_PR_209	PR	0.06			0.63		
1_M2_PR_357	PR	0.07			0.71		
1_M2_PR_356	PR	0.13			0.74		
1_M1_NE_045	NE	0.56	0.55	0.07	0.92	0.63	0.06
1_M1_NE_049	NE	0.45			0.81		
1_F1_NE_159	NE	0.45			0.81		
1_M2_NE_379	NE	0.43			0.80		
1_M2_NE_280	NE	0.13			0.40		
1_M1_CO_151	CO	0.00	0.63	0.12	0.12	0.89	0.07
1_M1_CO_148	CO	0.00			0.14		
1_F1_CO_270	CO	0.00			0.22		
1_F1_CO_273	CO	0.00			0.26		
1_M2_CO_395	CO	0.00			0.16		
1_M2_CO_396	CO	0.00			0.14		
1_M1_AN_063	AN	0.63	0.55	0.08	0.78	0.73	0.07
1_M1_AN_058	AN	0.35			0.39		
1_F1_AN_167	AN	0.60			0.82		
1_F1_AN_177	AN	0.55			0.92		
1_M2_AN_291	AN	0.88			0.99		
1_M2_AN_284	AN	0.78			0.96		
1_M1_FE_070	FE	0.38	0.11	0.02	0.54	0.30	0.03
1_M1_FE_071	FE	0.36			0.42		
1_F1_FE_181	FE	0.62			0.74		
1_F1_FE_182	FE	0.64			0.69		
1_M2_FE_305	FE	0.42			0.53		
1_M2_FE_304	FE	0.56			0.68		
1_M1_SA_090	SA	0.74	0.50	0.05	0.75	0.60	0.05
1_F1_SA_193	SA	0.39			0.61		
1_F1_SA_198	SA	0.72			0.82		
1_F1_SA_251	SA	0.38			0.41		
1_M2_SA_312	SA	0.40			0.50		
1_M2_SA_310	SA	0.64			0.72		
1_M1_DI_109	DI	0.68	0.00	0.00	0.63	0.17	0.02
1_M1_DI_100	DI	0.20			0.94		
1_F1_DI_170	DI	0.58			0.90		
1_M2_DI_328	DI	0.77			0.98		
1_M2_DI_334	DI	0.93			0.99		
1_M1_SH_146	SH	0.10	0.08	0.04	0.32	0.34	0.10
1_M1_SH_134	SH	0.13			0.31		
1_F1_SH_211	SH	0.02			0.18		
1_F1_SH_221	SH	0.11			0.29		
1_M2_SH_369	SH	0.13			0.42		
1_M2_SH_367	SH	0.15			0.28		
1_M1_GU_144	GU	0.09	0.40	0.07	0.38	0.75	0.09
1_F1_GU_220	GU	0.13			0.49		
1_M2_GU_364	GU	0.01			0.15		

## Appendix B

The accuracy data from the two validation tasks with children subjects are presented in Table A2.

**Table A2:** Proportion of correct responses in free naming task and emotion recognition task with predefined labels for each stimulus and averaged across emotion category. The data for the naming task is based on the answers of N = 32 children and the data for the recognition task is based on the answers of N = 28 children.

		Naming			Recognition		
Stimulus	Code	Pc Correct	M(Pc Correct)	SE(Pc Correct)	Pc Correct	M(Pc Correct)	SE(Pc Correct)
1_M1_HA_040	HA	0.81	0.87	0.01	0.79	0.90	0.03
1_M1_HA_056	HA	0.91			0.96		
1_M2_HA_282	HA	0.84			0.89		
1_M2_HA_281	HA	0.88			0.93		
1_F1_HA_161	HA	0.91			0.93		
1_F1_HA_162	HA	0.88			0.89		
1_MA_SU_094	SU	0.41	0.59	0.06	0.57	0.75	0.10
1_F1_SU_200	SU	0.63			0.79		
1_F1_SU_201	SU	0.44			0.64		
1_M2_SU_338	SU	0.25			0.82		
1_M2_SU_313	SU	0.59			0.50		
1_M1_PR_128	PR	0.09	0.22	0.04	0.21	0.42	0.06
1_M1_PR_130	PR	0.34			0.61		
1_F1_PR_208	PR	0.19			0.39		
1_F1_PR_209	PR	0.13			0.29		
1_M2_PR_357	PR	0.31			0.50		
1_M2_PR_356	PR	0.25			0.54		
1_M1_NE_045	NE	0.34	0.56	0.07	0.71	0.52	0.07
1_M1_NE_049	NE	0.44			0.75		
1_F1_NE_159	NE	0.38			0.64		
1_M2_NE_379	NE	0.38			0.68		
1_M2_NE_280	NE	0.06			0.39		
1_M1_CO_151	CO	0.00	0.39	0.06	0.04	0.74	0.06
1_M1_CO_148	CO	0.00			0.14		
1_F1_CO_270	CO	0.00			0.11		
1_F1_CO_273	CO	0.00			0.11		
1_M2_CO_395	CO	0.00			0.14		
1_M2_CO_396	CO	0.00			0.07		
1_M1_AN_063	AN	0.66	0.46	0.07	0.68	0.66	0.06
1_M1_AN_058	AN	0.31			0.29		
1_F1_AN_167	AN	0.56			0.86		
1_F1_AN_177	AN	0.63			0.86		
1_M2_AN_291	AN	0.75			0.93		
1_M2_AN_284	AN	0.63			0.89		
1_M1_FE_070	FE	0.50	0.17	0.03	0.50	0.27	0.02
1_M1_FE_071	FE	0.53			0.54		
1_F1_FE_181	FE	0.72			0.64		
1_F1_FE_182	FE	0.56			0.57		
1_M2_FE_305	FE	0.53			0.54		
1_M2_FE_304	FE	0.44			0.64		
1_M1_SA_090	SA	0.75	0.55	0.04	0.68	0.57	0.02
1_F1_SA_193	SA	0.50			0.50		
1_F1_SA_198	SA	0.72			0.64		
1_F1_SA_251	SA	0.47			0.21		
1_M2_SA_312	SA	0.28			0.50		
1_M2_SA_310	SA	0.63			0.57		
1_M1_DI_109	DI	0.44	0.00	0.00	0.57	0.10	0.02
1_M1_DI_100	DI	0.25			0.68		
1_F1_DI_170	DI	0.28			0.68		
1_M2_DI_328	DI	0.44			0.89		
1_M2_DI_334	DI	0.56			0.89		
1_M1_SH_146	SH	0.13	0.19	0.07	0.29	0.31	0.08
1_M1_SH_134	SH	0.16			0.36		
1_F1_SH_211	SH	0.03			0.21		
1_F1_SH_221	SH	0.19			0.29		
1_M2_SH_369	SH	0.28			0.21		
1_M2_SH_367	SH	0.22			0.25		
1_M1_GU_144	GU	0.28	0.32	0.07	0.21	0.64	0.06
1_F1_GU_220	GU	0.22			0.46		
1_M2_GU_364	GU	0.06			0.25		

## Appendix C

The confusion matrices of the emotion stimuli and all possible responses in the emotion recognition task with adult and child participants are shown in Table A3 and Table A4, respectively.

**Table A3:** Confusion matrix of the responses in the emotion recognition task of N = 104 adult participants ^1^.

Adults		Presented Stimuli										
		HA	SU	PR	NE	CO	AN	FE	SA	DI	SH	GU
Responses	**HA**	87%	22%	22%	0%	0%	0%	0%	0%	1%	0%	0%
	**SU**	0%	73%	0%	0%	1%	1%	35%	0%	0%	3%	0%
	**PR**	5%	2%	67%	2%	1%	1%	0%	2%	0%	0%	1%
	**NE**	8%	0%	10%	75%	8%	0%	0%	1%	0%	2%	2%
	**CO**	0%	0%	0%	3%	18%	1%	1%	9%	0%	5%	5%
	**AN**	0%	0%	0%	2%	1%	81%	0%	8%	6%	0%	5%
	**FE**	0%	2%	0%	4%	3%	1%	60%	1%	0%	2%	1%
	**SA**	0%	0%	0%	5%	37%	3%	0%	65%	1%	11%	27%
	**DI**	0%	0%	0%	0%	1%	5%	1%	2%	89%	1%	1%
	**SH**	0%	0%	0%	3%	10%	2%	0%	4%	1%	30%	24%
	**GU**	0%	0%	0%	3%	20%	5%	2%	7%	1%	47%	35%

^1^ The basic emotions are highlighted in gray. Each column represents 1 of the 11 presented emotional categories. Each column represents the responses out of the 11 possible labels. The basic emotional expressions are highlighted in gray.

**Table A4:** Confusion matrix of the responses in the emotion recognition task of N = 28 child participants ^1^.

Children		Presented Stimuli										
		HA	SU	PR	NE	CO	AN	FE	SA	DI	SH	GU
Responses	**HA**	90%	25%	44%	1%	1%	0%	2%	1%	1%	1%	0%
	**SU**	2%	66%	4%	1%	1%	1%	31%	0%	0%	5%	0%
	**PR**	4%	4%	42%	4%	2%	0%	0%	2%	0%	0%	0%
	**NE**	3%	1%	7%	64%	7%	1%	0%	4%	0%	10%	5%
	**CO**	0%	0%	1%	4%	10%	1%	1%	3%	0%	3%	7%
	**AN**	0%	0%	0%	2%	7%	75%	0%	10%	18%	2%	14%
	**FE**	0%	4%	1%	3%	7%	1%	57%	1%	0%	1%	1%
	**SA**	0%	0%	0%	6%	29%	2%	2%	52%	0%	14%	17%
	**DI**	0%	0%	1%	1%	0%	5%	2%	6%	74%	2%	2%
	**SH**	1%	0%	1%	13%	15%	4%	1%	5%	3%	27%	23%
	**GU**	0%	0%	0%	3%	21%	10%	4%	17%	4%	36%	31%

^1^ The basic emotions are highlighted in gray. Each column represents 1 of the 11 presented emotional categories. Each column represents the responses out of the 11 possible labels. The basic emotional expressions are highlighted in gray.
